# Ethanol-Induced Mitochondrial Damage in Sertoli Cells is Associated with Parkin Overexpression and Activation of Mitophagy

**DOI:** 10.3390/cells8030283

**Published:** 2019-03-25

**Authors:** Nabil Eid, Yuko Ito, Akio Horibe, Yoshinori Otsuki, Yoichi Kondo

**Affiliations:** 1 Department of Anatomy and Cell Biology, Division of Life Sciences, Osaka Medical College, 2-7 Daigaku-machi, Takatsuki, Osaka 569-8686, Japan; an1006@osaka-med.ac.jp (Y.I.); konchan@osaka-med.ac.jp (Y.K.); 2 Kubomizuki lady’s clinic 3-13-8, Mikatadai, Nishi-ku, Kobe, Hyogo 651-2277, Japan; horibe@kubomizuki.or.jp; 3 Osaka Medical College, 2-7 Daigaku-machi, Takatsuki, Osaka, 569-8686, Japan; y.otsuki@osaka-med.ac.jp

**Keywords:** ethanol, mitochondria, autophagy, LC3, apoptosis, Sertoli cell, Parkin, PINK1, TFEB, mitophagy, infertility

## Abstract

This study was conducted to elucidate the involvement of the PINK1-Parkin pathway in ethanol-induced mitophagy among Sertoli cells (SCs). In the research, adult rats were given intraperitoneal injections of ethanol (5 gm/kg) and sacrificed at various time periods within 24 h. Transmission electron microscopy was applied to reveal enhanced mitochondrial damage in SCs of the ethanol-treated rats (ETRs) in association with a significant increase in numbers of mitophagic vacuoles (mitophagosomes and autolysosomes) in contrast to very low levels in a control group treated with phosphate-buffered saline (PBS). This enhancement was ultra-structurally verified via observation of trapped mitochondria within LC3-labeled membranes, upregulation of LC3 protein levels, colocalization of LC3 and cytochrome c, and reduced expression of mitochondrial proteins. Importantly, Parkin expression was found to be upregulated in ETR SCs, specifically in mitochondria and mitophagosomes in addition to colocalization with PINK1 and pan-cathepsin, indicating augmented mitophagy. Transcription factor EB (TFEB, a transcription factor for autophagy and mitophagy proteins) was also found to be upregulated in nuclei of ETR SCs and associated with enhanced expression of iNOS. Enhanced Parkin-related mitophagy in ETR SCs may be a protective mechanism with therapeutic implications. To the authors’ knowledge, this is the first report demonstrating the ultrastructural characteristics and molecular mechanisms of Parkin-related mitophagy in ETR SCs.

## 1. Introduction

Autophagy (or macroautophagy) is a catabolic pathway for lysosomal degradation of most cellular components under basal conditions and upon exposure to various stressors such as starvation, oxidative/nitrosative stress, mitochondrial damage, and lipogenic challenge [1,2,3]. Selective autophagic removal of damaged mitochondria, or mitophagy, is an antiapoptotic mechanism induced and specifically upregulated as a response to various damaging agents such as protonophore carbonyl cyanide m-chlorophenyl hydrazine (or CCCP; used for in vitro studies) and ethanol in animal models [4,5,6]. The ultrastructural characteristics of mitophagy in hepatocytes [6,7] and Sertoli cells (SCs) [8,9] of acute ethanol-treated rats (an animal model representing binge-type exposure to ethanol) were recently reported by the authors’ laboratory. These include the engulfment of damaged mitochondria by microtubule-associated protein 1 light chain3 (LC3)-mediated autophagosomal membranes forming mitophagosomes that fuse with lysosomes, creating autolysosomes with perinuclear localization. The PINK1/Parkin mitophagic pathway is characterized by the interplay of two recessive Parkinson’s-linked genes (PTEN-induced kinase 1 (PINK1) and Parkin (an E3 ubiquitin ligase), which maintain mitochondrial homeostasis and clear dysfunctional mitochondria via mitophagy. Mutations affecting PINK1-Parkin genes cause Parkinson’s disease (PD). The specific molecular mechanisms of ethanol-induced hepatic mitophagy were recently reported to be related to the PINK1-Parkin pathway [6,7,10,11,12,13]. In these studies, ethanol-induced mitochondrial damage via mechanisms related to mitochondrial DNA (mt DNA) damage, oxidative stress, and other factors caused the stabilization of PINK1 (a sensor of mitochondrial damage) on damaged mitochondria. This results in Parkin (a specific marker of mitophagy) overexpression and translocation to damaged mitochondria, protein ubiquitination and subsequent mitochondrial fragmentation, and engulfment of mitochondria by LC3-mediated autophagosomal membranes. The pro-survival role of Parkin against ethanol toxicity has recently been reported in a few studies. In ethanol-treated Parkin knock-out (KO) mice, there was a reduction of mitophagy leading to increased hepatocyte damage and steatosis [12,13]. Parkin deficiency has been found to exacerbate ethanol-induced dopaminergic neurodegeneration in mice via the reduction of anti-apoptotic mitophagy [14]; on the other hand, Parkin overexpression protected retinal ganglion cells via mitophagy activation in an experimental glaucoma rat model [15].

Although SCs play essential roles for germ cell survival and fertility maintenance in response to toxic insults such as binge-type ethanol exposure [16], no studies investigating the mitophagy-related PINK1-Parkin pathway in SCs have yet been reported. In this study, the authors investigated the ultrastructural characteristics and specific molecular mechanisms of ethanol-induced mitophagy in SCs of acute ETRs and the involvement of the PINK1-Parkin pathway as well as associated transcription factor EB (TFEB) (a master transcription factor for autophagy and Parkin-related mitophagy) [16,17,18]. Light and electron microscopic techniques along with Western blot analysis showed evidences of upregulation and mitochondrial translocation of Parkin and PINK1 among ETR SCs in association with the formation of LC-3 mediated mitophagosomes and nuclear translocation of TFEB.

## 2. Materials and Methods

### 2.1. Study Approval

Twelve adult male rats (10 weeks old) with an approximate average weight of 300 g were purchased from SLC Japan Co. (Shizuoka, Japan). They were treated in keeping with the relevant Experimental Animal Research Committee of Osaka Medical College guidelines (approved by Animal Research Committee of Osaka Medical College on 10/28/2013, under code, 25090).

### 2.2. Antibodies and Kits

The following primary antibodies were used: Rabbit anti-LC3B antibody (PM063) from MBL, Nagoya, Japan; rabbit anti-PINK1 (BC100-494), rabbit anti-Parkin (NB100-91921), and mouse anti-p62 (H00008878-M01) antibodies from Novus Biologicals (Briarwood Avenue, Building IV Centennial, CO, USA); goat anti-pan-cathepsin (sc-6499), mouse anti-Parkin (sc-32282), mouse anti-Actin (sc-47778), and mouse anti-cytochrome c (7H8):(sc-13560) antibodies from Santa Cruz Biologicals (Dallas, TX, USA); rabbit anti-inducible nitric oxide synthase (iNOS) ( ab15326 ) and rabbit anti-iNOS (ab15323) antibodies from Abcam Biologicals (Cambridge, MA, USA); rabbit anti-TFEB (MBS9125929) from MyBioSource Biologicals (San Diego, CA, USA); and rabbit anti-Cytochrome c oxidase (COX) IV (3E11) from cell signaling Biologicals (Danvers, MA, USA). Alexa Fluor 488- or 594-conjugated secondary antibodies (Molecular Probes, Carlsbad, CA, USA) and VectaFluor™ R.T.U. Antibody Kit DyLight^®^ 488 were used for immunofluorescence (IF) studies (Vector, CA, USA), while 4′,6-diamidino-2-phenylindole (DAPI) (H-1200) (vector) was used for nuclear counterstaining. A TUNEL kit (Roche Diagnostics, Mannheim, Germany) was used for apoptosis detection. Vectastain ABC Standard Kit (PK-4000) and ImmPACT DAB(SK-4105) from Vector were used for immunohistochemistry (IHC). Donkey anti-rabbit IgG-HRP (sc-2077) and donkey anti-mouse IgG-HRP (SC-2096) secondary antibodies from Santa Cruz were used for Western blot. A total of 15 nm and 6 nm gold-conjugated goat anti-rabbit and anti-mouse antibodies, respectively (Aurion, Wageningen, The Netherlands), were used for immunoelectron microscopy (IEM). As a rule, we followed manufacturer’s protocols and our previous publications regarding the use of antibodies and kits in Western blot, IF, IHC, and IEM.

### 2.3. Animals and Experimental Procedure

The animals were treated with a single 5 g/kg intraperitoneal dose of ethanol (40% v/v) consistent with animal models of binge ethanol exposure [6,16,19,20]. A control group received equal volume of phosphate buffered saline (PBS). Following ethanol administration, the rats were sacrificed by cervical dislocation at various time points (0, 3, 6, and 24 h). For paraffin embedding, the testes were divided into small pieces and fixed in 4% paraformaldehyde. Some testicular pieces were fixed in 2% paraformaldehyde and 2.5% glutaraldehyde in 0.1 M phosphate buffer for embedding in epoxy and observation under transmission electron microscopy (TEM) as we previously reported [6,8,16]. Fresh samples were frozen in liquid nitrogen for Western blot analysis consistent with our earlier study [21].

### 2.4. IHC for LC3, Parkin, PINK1, TFEB, and iNOS 

The immunohistochemical labeling methods were performed according to the manufacturer’s recommendations and our recent studies [6,18,21,22]. Paraffin-embedded sections (4-µm thickness) underwent a deparaffinization process, antigen retrieval, blocking of endogenous peroxidase activity, and non-specific antigen binding. The sections were then incubated for 1 hour at room temperature with the primary antibodies mentioned above. Immunostaining was performed by Vectastain ABC method. Then, sections were treated with DAB, counterstained with hematoxylin, and observed under Olympus BX41microscope (BX41, Olympus, Tokyo, Japan). Quantification of LC3, TFEB, and iNOS immunostaining in SCs was performed on 10–15 seminiferous tubules from ETRs and the control group. Using Adobe Photoshop, the tubules were captured and saved for computer analysis using Image J (National Institutes of Health, Bethesda, MA, USA). The intensity of protein expression in SCs was quantified as recently reported [22].

### 2.5. IF Single and Double Labeling of Mitophagy Proteins and Mitochondrial and Lysosomal Markers

IF labelling of TFEB was performed on paraffin sections as in IHC. In brief, after deparaffinization, antigen retrieval, and serum blocking, TFEB antibody was applied for 1 h at room temperature. The sections were incubated with Alexa Fluor 594-conjugated secondary antibody for 30 min. For double labeling of LC3 with either cytochrome c (a mitochondrial marker) or p62, we used a simultaneous application of two primary antibodies followed by Alexa Fluor 594 and 488-conjugated secondary antibodies [6,23,24]. For double labeling of pan-cathepsin (lysosomal marker) with either Parkin or LC3, we used a sequential method as previously reported [6,16,21,22]. In brief, following incubation with primary antibodies for 1 hour, Alexa Fluor 594 and VectaFluor™ R.T.U. DyLight^®^ 488 were used as secondary reagents (30 minutes). After nuclear counterstaining with DAPI (blue reaction), the sections were observed under the BX41 fluorescence microscope.

### 2.6. Line Profile Plots for Co-Localization Analysis of Parkin and Pan-Cathepsin

Line profiles from the two fluorescent channels were analyzed using Image J software as reported (6,23,24). Line profile plots reflect intensity and colocalization of two different proteins as overlapped red and green peaks (vertical axis shows intensity of fluorescence while horizontal axis indicates distance). 

### 2.7. Terminal Deoxynucleotidyl Transferase dUTP-Mediated Nick-End Labeling (TUNEL) Assay 

TUNEL assay for apoptosis detection was performed as previously reported [6,16]. Deparaffinized sections were treated with TUNEL reaction mixture (TdT enzyme and fluorescent-labeled nucleotides) for 1 h at 37 °C. TUNEL positive cells showed green labeling under fluorescence microscope, while TUNEL negative nuclei appeared blue with DAPI. 

### 2.8. TEM and Quantitative Analysis of Mitophagic Vacuoles (MVs)

Ultrathin sections at 70 nm thickness were cut with a diamond knife, double-stained with uranyl acetate and lead citrate, and examined under an H-7650 transmission electron microscope (Hitachi, Tokyo, Japan). For quantification of MVs (mitophagosomes and autolysosomes) in SCs, 15–20 lower magnification photomicrographs from the testes of controls and ETRs (×2500 magnification, each image containing at least a portion of SC nucleus showing the perinuclear area) were used as described previously [6,8,16,25].

### 2.9. Immunogold Labeling for LC3, Parkin, and TFEB and Double Immunogold Labeling of Parkin and PINK1

The method of post embedding immune-gold labeling was based on our previous reports [6,7,26]. Ultrathin sections mounted on nickel grids were etched with either 5% sodium metaperiodate for 15 minutes [27] or 1%–2% H202 for 10 minutes [28]. The sections were then washed in filtered water and incubated in 3% BSA in PBS for 1 h. After incubation with the same primary for Parkin, LC3, and TFEB for 2 h at room temperature, the sections were incubated with 15-nm gold-conjugated goat anti-rabbit secondary antibody according to instructions of the producing company. For double immunogold labeling of Parkin and PINK1, a mixture of these antibodies was simultaneously applied for 2 h followed by a mixture of gold-conjugated goat anti-mouse (6 nm) and gold-conjugated goat anti-rabbit (15 nm) secondary antibodies for 1 h. Grids were washed and briefly stained briefly with uranyl acetate and lead citrate. For quantification of LC3 and Parkin immunogold particles (15 nm) in control and ETRs SCs, a total of 15–20 mitochondria from each group were selected and immunogold particles for each protein were counted [29]. Mitochondria with double PINK1 (15 nm) and Parkin (6 nm) immunogold labeling were identified and counted using 10–15 higher magnification photomicrographs from the testes of controls and ETRs. Quantification of nuclear TFEB immunogold particles was performed on 15–20 images from each group (each image containing at least a portion of SC nucleus). The Student *t*-test was used to assess the statistical significance of all these quantifications.

### 2.10. Western Blot Analysis for LC3, Cytochrome c, Parkin, TFEB, COX IV, and iNOS

After homogenization of whole testicular tissues in a modulated RIPA buffer followed by centrifugation, the supernatant was electrophoresed on 12% sodium dodecyl sulfate polyacrylamide gel and transferred onto a polyvinylidene difluoride membrane. Proteins were detected with the specific primary antibodies for LC3, cytochrome c, Parkin, TFEB, COX IV, and iNOS, and then with specific peroxidase-labeled secondary antibodies as previously reported [6,21,22]. The relative intensity of expression of various proteins against actin (43 kDa) was normalized and densitometrically measured using Image J.

### 2.11. Statistical Analysis

Statistical analysis was performed by GraphPad Prism 8 Software (8.0.2), San Diego, CA, USA. Differences between more than two groups were tested by analysis of variance (ANOVA) with *p* < 0.05 considered as statistically significant. The Student *t*-test was used for comparison between two groups.

## 3. Results

### 3.1. Enhanced Mitochondrial Damage and Mitophagic Vacuole (MV) Formation in ETR SCs with Predominant Localization in Perinuclear Areas

**Figure 1:**
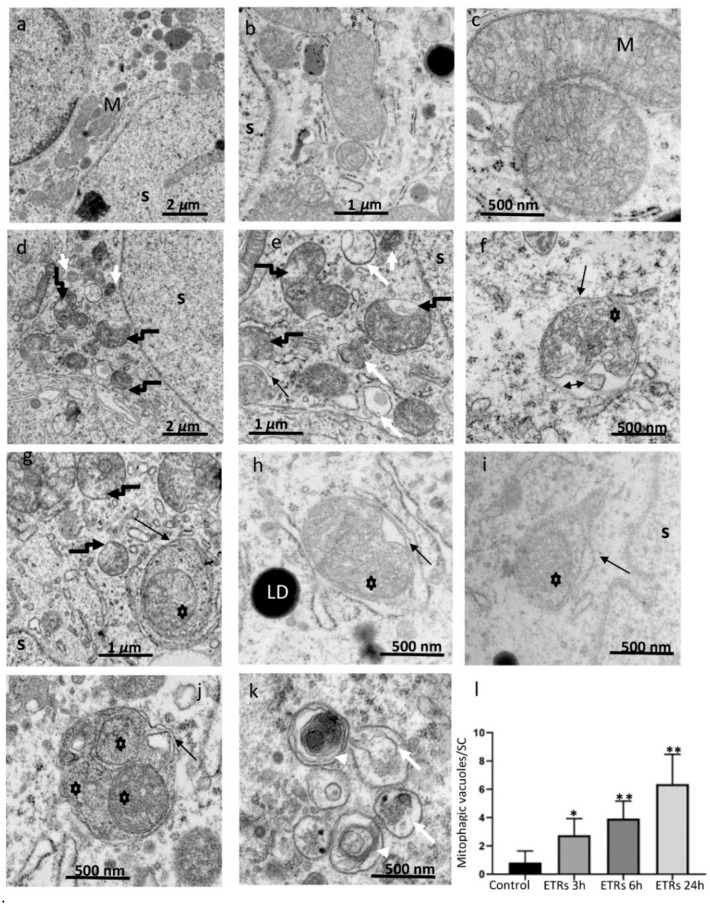
Ultrastructural characteristics of enhanced mitochondrial damage and mitophagy in ethanol-treated rats (ETR) Sertoli cells (SCs.) (**a**–**c**): control testes; (**d**–**k**): ETRs. Quantification of mitophagy is shown in (**l**). Note the normal mitochondria (M) in control testes with characteristic vesicular-type cristae. Broken black arrows (**d,e,g**) indicate damaged mitochondria in ETR SCs, while black arrows show autophagosomal membranes engulfing damaged mitochondria (asterisks) forming mitophagosomes. The double-head arrow indicates damaged fragmented cristae. The long and short white arrows mark autolysosomes and lysosomes, respectively. White arrow heads mark multilamellar bodies. LD: lipid droplets; S: SC nucleus. The histogram depicts quantification of mitophagic vacuoles in the control and ETRs. * *p* < 0.01 and ** *p* < 0.001 vs. control (one-way analysis of variance (ANOVA)).

The animals were subjected to a single injection of ethanol (5 g/kg) or PBS (for the control group) and sacrificed at 0, 3, 6, and 24 h after injection (following a model of acute alcohol toxicity) [6,16,19,20]. As shown in Figure 1a–c, while SCs in the control group exhibited normal mitochondrial morphology (a smooth outer membrane and an inner membrane contiguous with a vesicular type of cristae and containing a granular, moderately electron-dense internal matrix) and distribution over the whole cytoplasm, the mitochondria in ETR SCs (Figure 1d–f) showed perinuclear aggregation with damaged or lost cristae and a dark matrix, in addition to fragmentation, along with outer-membrane irregularities. The damaged mitochondria were associated with MVs including mitophagosomes (Figure 1g–j) and autolysosomes (mitophagolysosomes) (Figure 1e,k). Multilamellar bodies (Figure 1k) were also frequently observed, indicating enhanced mitochondrial damage [6,16,26]. This juxtanuclear accumulation of MVs in ETR SCs is shown with low-power magnification (Figure S1). Importantly, based on TEM and TUNEL (Figure S2), germ cell apoptosis was frequently observed in ETR testes, but SCs nuclei appeared normal. This indicates that the enhanced mitophagic response may be anti-apoptotic in nature [6,16]. Quantitative analysis (Figure 1l) and control-group comparison revealed a significant increase in MV formation for all time periods after ethanol injection, with a peak at 24 h. With this in mind, the 24-h time point was chosen for analysis in subsequent experiments. As double-layered membranes in mitophagosomes indicate the involvement of the LC3-related autophagic mechanism [6,16,22,26], the expression of this protein was investigated.

### 3.2. Association of Ethanol-Induced Mitophagosomes in SCs with Increased LC3-II Expression and Mitochondrial Proteins Reduction

**Figure 2:**
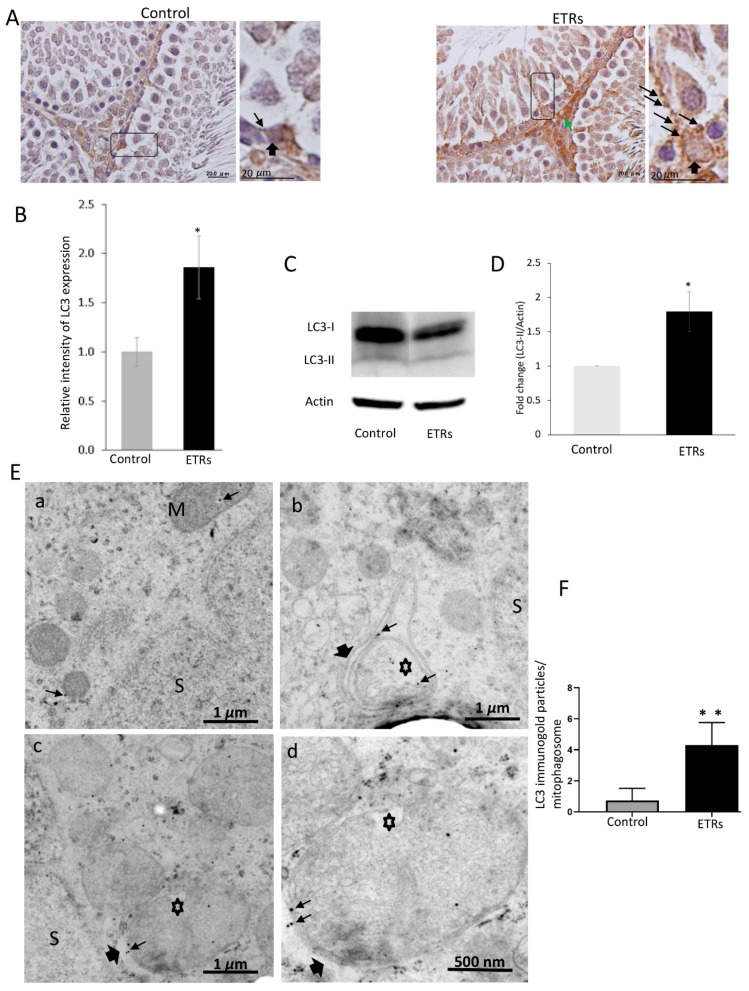
Enhanced light chain3 (LC3)-II expression in ETR SCs with specific localization to mitophagosomes (**A**) immunohistochemistry (IHC) of LC3. The framed areas are magnified in the insets on the right. LC3-II puncta are marked by long thin arrows, while short thick arrows indicate SCs nuclei. The green arrow marks expression in interstitial cells. (**B**) Quantification of LC3 expression in control and ETR SCs. (**C**) Western blot of LC3. The relative expression level of the protein is normalized to actin and expressed as a fold of the control (*n* = 3) (this normalization applies to blots of other proteins shown below.) (**D**) Histogram showing significant increase of LC3-II in ETRs compared to the control. *P* < 0.05 (*t*-test). (**E**) LC3 immunogold labeling in the control (**a**) and ETR SCs (**b**–**d**). Thin arrows indicate LC3 immunogold particles, while thick arrows mark autophagosomal membranes sequestering damaged mitochondria (asterisks) forming mitophagosomes. (**F**) Histogram demonstrating significant increase in the number of LC3-II immunogold particles in mitophagosomes of ETRs SCs compared to the control group. *P* < 0.01 (*t*-test). M: normal mitochondria; S: SC nucleus.

**Figure 3:**
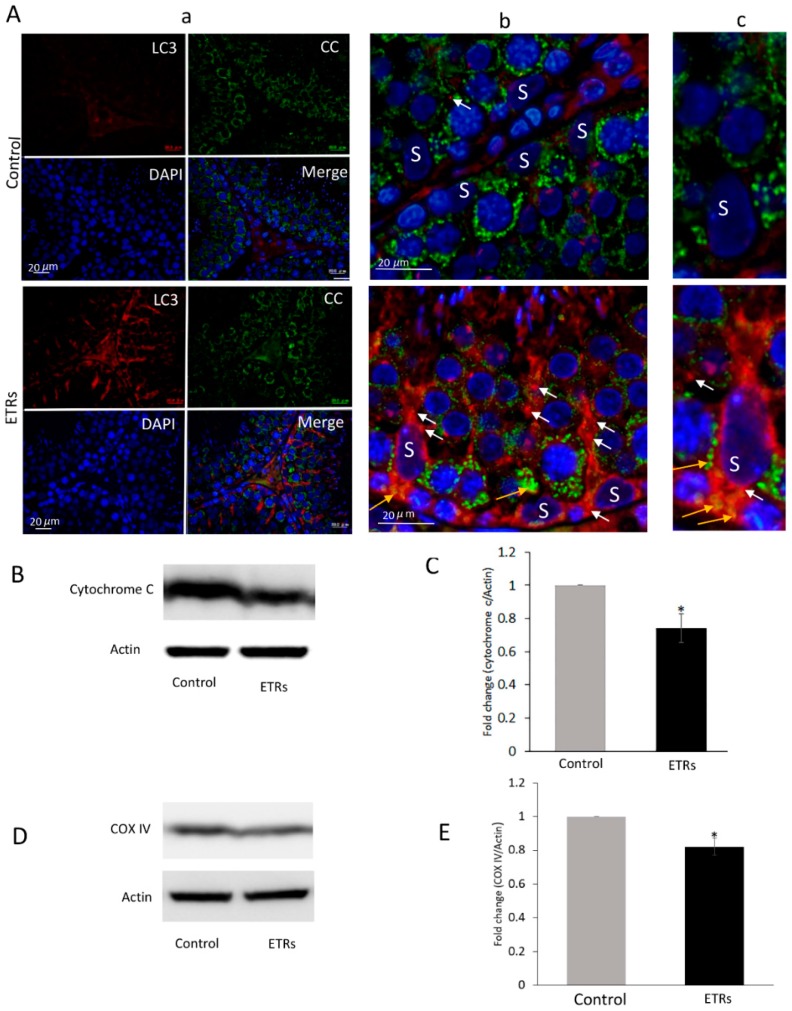
Enhanced colocalization of LC3 with cytochrome c in SCs of ETRs and reduced expression of mitochondrial proteins. (**A**) Immunofluorescence (IF) double-labeling of LC3 (red) and cytochrome c (CC) (green) with nuclear counterstaining using DAPI (blue). The panels in the middle (**b**) and on the right (**c**) are higher magnifications of those on the left in (**a**). Orange arrows indicate areas of colocalization for proteins (yellow-orange), while white arrows indicate LC3 puncta. S: SC nucleus. (**B**) Western blot of CC (*n* = 3). The histogram in (**C)** shows significant reduction of CC in ETRs. (**D**) Western bot of Cytochrome c oxidase (COX) IV (*n* = 3). The histogram in **E** shows significant reduction of the protein in ETRs * *p* < 0.05 (*t*-test).

The IHC characteristic in Figure 2A clearly demonstrates enhanced formation of LC3 puncta (indicating the induction of LC3-II isoform required for maturation of autophagosomal membrane) in ETR SCs compared to very low levels in the control group, indicating elevated mitophagosome formation (mediated by LC3-II) as previously reported by other authors [6,16,22,26] and in line with the TEM findings detailed in Figure 1. Increased LC3 expression was also observed in interstitial cells of ETRs. Quantitative analysis of LC3 expression in SCs (Figure 2B) demonstrated higher LC3 intensity in ETRs SCs compared to control group, which was statistically significant. As also shown in Figure 2C,D, Western blot analysis indicated the upregulation of LC3-II (16 kDa), supporting the findings made from light-microscope observation. IF double-labeling of LC3 and P62 (an LC3 adaptor molecule) showed enhanced co-localization in ETR SCs, thereby confirming enhanced mitophagic response (Figure S3) [30]. Immunoelectron microscopy (IEM; Figure 2E) demonstrated a very low presence of LC3-II immunogold particles in control SCs. However, a significant increase in LC3-II immunogold labeling (Figure 2F) was observed within mitophagosomes on autophagosomal membranes in ETR SCs, indicating the autophagic nature of mitophagosomes [21,26]. Double-labeling with IF LC3 and cytochrome c revealed enhanced co-localization in ETR SCs (Figure 3A), indicating the formation of mitophagosomes as reported in past studies [6,10,11,23,24]. Western blot and analysis (Figure 3B,C) showed a significant reduction of cytochrome c (15 kDa), indicating mitochondrial damage within MVs [10,11,23,24,31]. Additional Western blot analysis showed that the expression level of COX IV (17 kDa) (inner mitochondrial membrane protein) was also reduced in testes of ETRs (Figure 2D,E). Against this background, Parkin expression was investigated to help identify the specific proteins potentially involved in enhanced MV formation in ETR SCs.

### 3.3. Ethanol Increased the Expression and Mitochondrial Translocation of Parkin and PINK1 in SCs and Fusion with Lysosomal Compartment 

**Figure 4:**
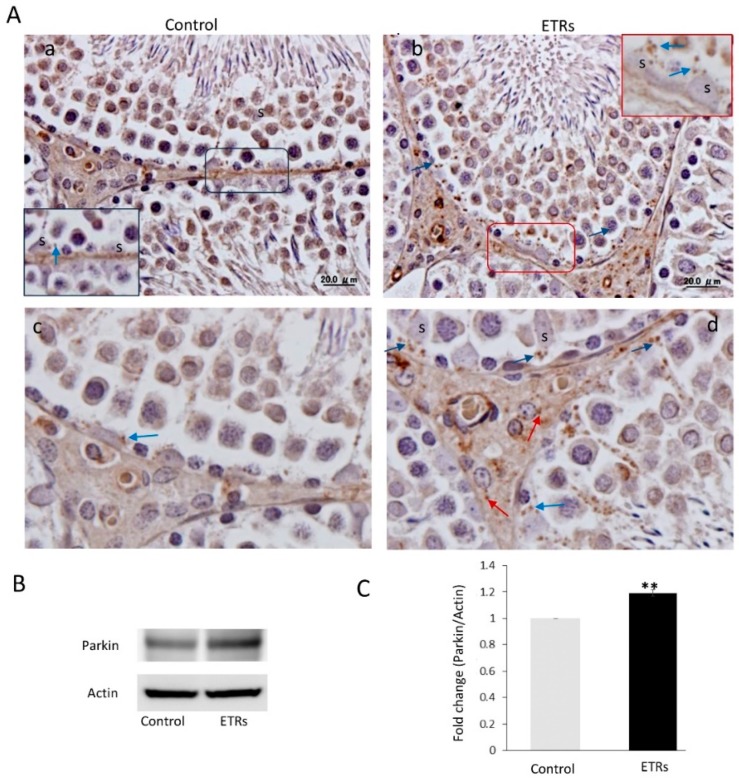
Ethanol-related increase in Parkin expression in SCs with predominant perinuclear localization (**A**) IHC of Parkin in the control (**a**) and ETRs (**b**). The insets are higher magnifications of the framed areas showing Parkin expression (blue arrows). The lower panels (**c**,**d**) are higher magnifications demonstrating Parkin expression in SCs and Leydig cells (red arrows). S: SC nuclei. (**B**) Western blot of Parkin (*n* = 3). The histogram in (**C)** shows a significant increase in Parkin expression in ETRs. ** *p* < 0.01 (*t*-test).

**Figure 5:**
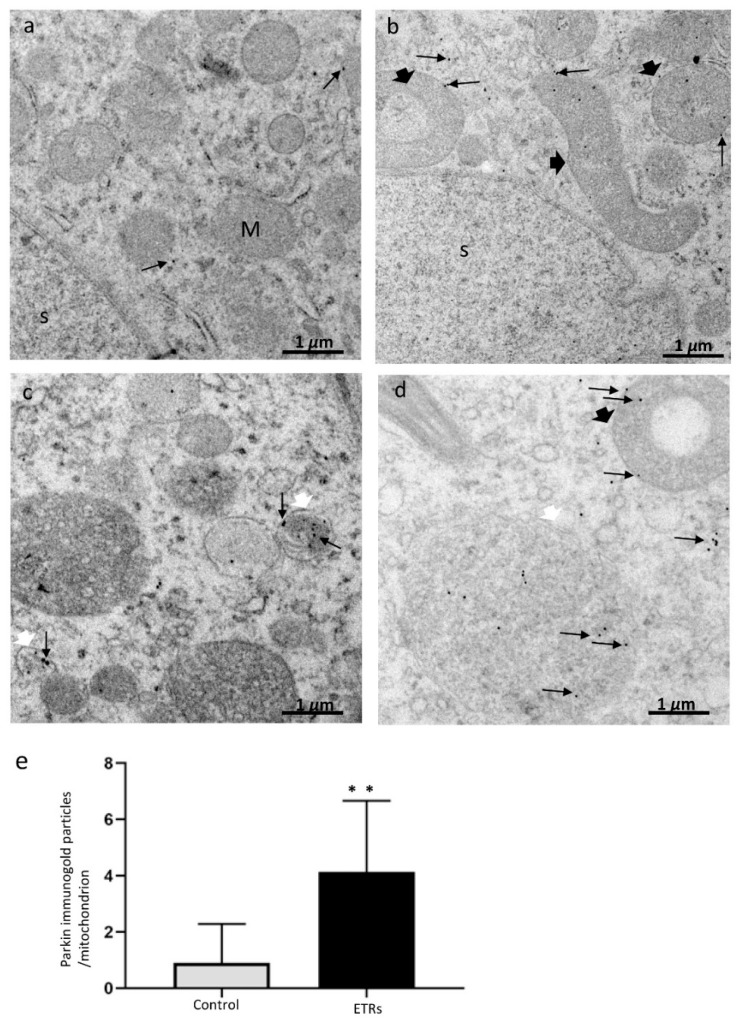
Ultrastructural characteristics of Parkin overexpression and mitochondrial translocation in ETR SCs. (**a**) control; (**b**–**d**) ETRs. The long black arrows mark Parkin immunogold particles, while the short black arrows indicate damaged mitochondria. The white arrows mark the autophagosomal membrane. S: SC nucleus; M: normal mitochondria. (**e**) Histogram demonstrating significant increase in the number of Parkin immunogold particles in the mitochondria of ETRs SCs compared to control. ** *p* < 0.01 (*t*-test).

**Figure 6:**
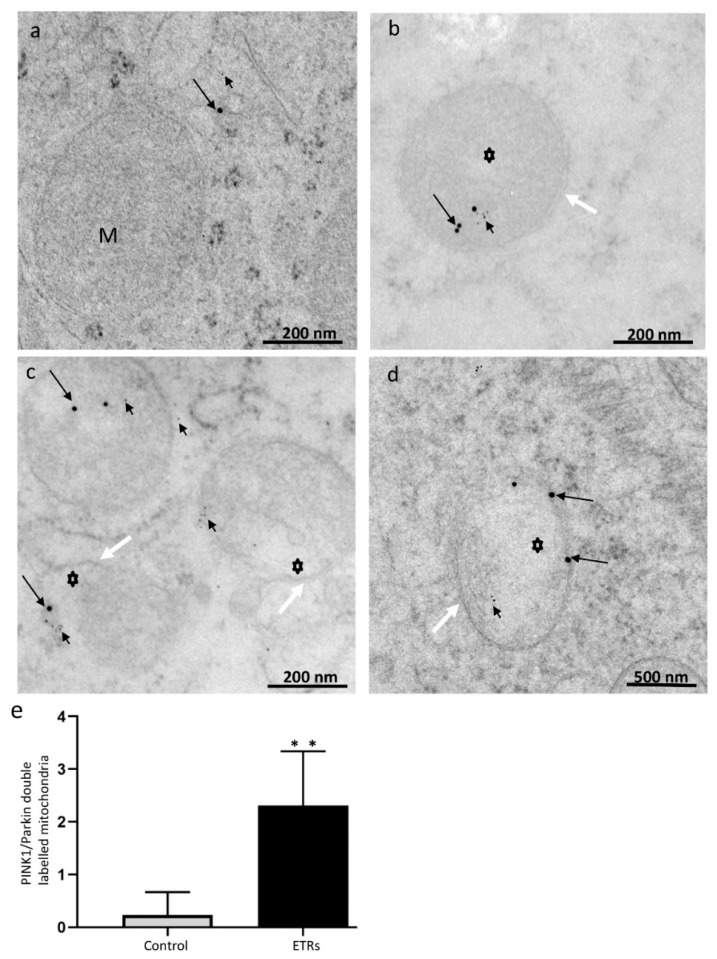
Immunogold double labeling of PINK1 and Parkin in control (a) and ETR SCs (b–d). The long black arrows indicate PINK1 (labeled with 15 nm gold particles), while short black arrows show Parkin (6 nm gold particles) localizations to damaged mitochondria (asterisks) of ETRs. The white arrows indicate autophagosomal membranes. M, mitochondria; S, SC nucleus. (e) Histogram showing quantification of mitochondria positive for both PINK1 and Parkin. ** *p* < 0.01 (*t*-test).

**Figure 7:**
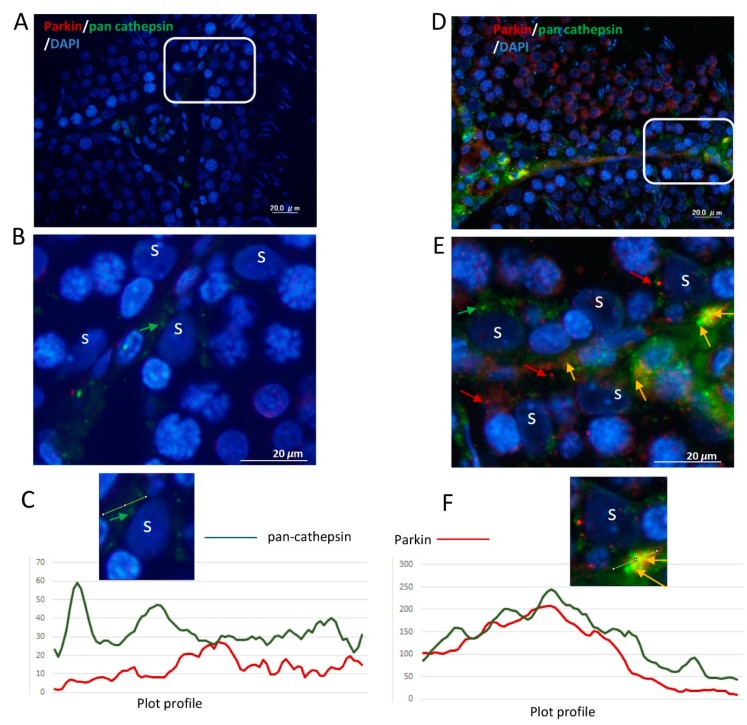
Elevated expression and colocalization of Parkin with pan cathepsin in ETRs SCs. (control, **A**–**C**), (ETRs, **D**–**F**). The framed areas in (A) and (D) are magnified in (**B)** and (**E)**, respectively. Note the enhanced expression and colocalization of Parkin (red, red arrows) and pan-cathepsin (green, green arrows) upon merging (orange-yellow, orange arrows) in ETR SCs (D,E) compared to control (A,B). S: SC nucleus. DAPI (blue) was used for nuclear counterstaining. (**C**,F) Plot profiles demonstrating the colocalization of Parkin and pan-cathepsin defined as overlapped red and green peak at the lines positioned in images (cropped from panels B,E) just above the histograms.

As shown in Figure 4A–C, IHC showed enhanced expression of Parkin in SCs and Leydig cells of ETRs as compared to lower levels in control testes. A low level of expression was also observed in mature sperm (data not shown). Parkin expression was specifically perinuclear, which was consistent with TEM findings for enhanced MV formation (Figure 1). Consistent with IHC, Western blotting and analysis revealed significant upregulation of Parkin (52 kDa) in ETRs testes. As mitochondrial translocation of Parkin is essential for recognition and clearance of damaged mitochondria by LC3-II-mediated autophagic membranes [6,7,10,11,12,13], the authors studied the subcellular localization of Parkin using IEM (Figure 5). As shown in this figure and in consistency with the immunogold labeling of LC3-II in mitophagosomes (Figure 2E), Parkin-immunogold particles were observed in damaged mitochondria and mitophagosomes in ETR SCs in contrast to very low levels in the control. This supports the authors’ recent liver research using the same animal mode [6,7]. As PINK1 accumulation in damaged mitochondria is required for Parkin mitochondrial translocation [6,7,10,11,26], the co-expression of PINK1 and Parkin at the ultrastructural level was investigated. As expected, PINK1 and Parkin immunogold particle presence was increased in damaged mitochondria and mitophagosomes in ETR SCs (Figure 6b–d), while signals were very weak in the control group (Figure 6a). Statistical analysis (Figure 6e) revealed significant increase in the number of mitochondria positive for both PINK1-Parkin immunogold labeling in ETRs SCs compared to control group. This accumulation of PINK1 was verified by IHC (Figure S4). To check the degradation of mitophagosomes via the lysosomal system, double-labeling of Parkin with pan-cathepsin was also performed. As shown in Figure 7, the expression levels and colocalization signals of Parkin with pan-cathepsin in ETR SCs (Figure 7D,E) were higher than control group (Figure 7A,B), indicating enhanced mitophagic activity and formation of mitophagolysosomes [10]. These findings were confirmed by plot profile analysis (Figure 7C,F) [6,7,23,24] and LC3 and pan-cathepsin double-labeling (Figure S5), thereby supporting the authors’ previous findings, indicating the acceleration of autophagic flux [6,16]. 

### 3.4. Enhanced Mitophagy in SCs of ETRs is Associated with TFEB Nuclear Translocation and Induction of iNOS

**Figure 8:**
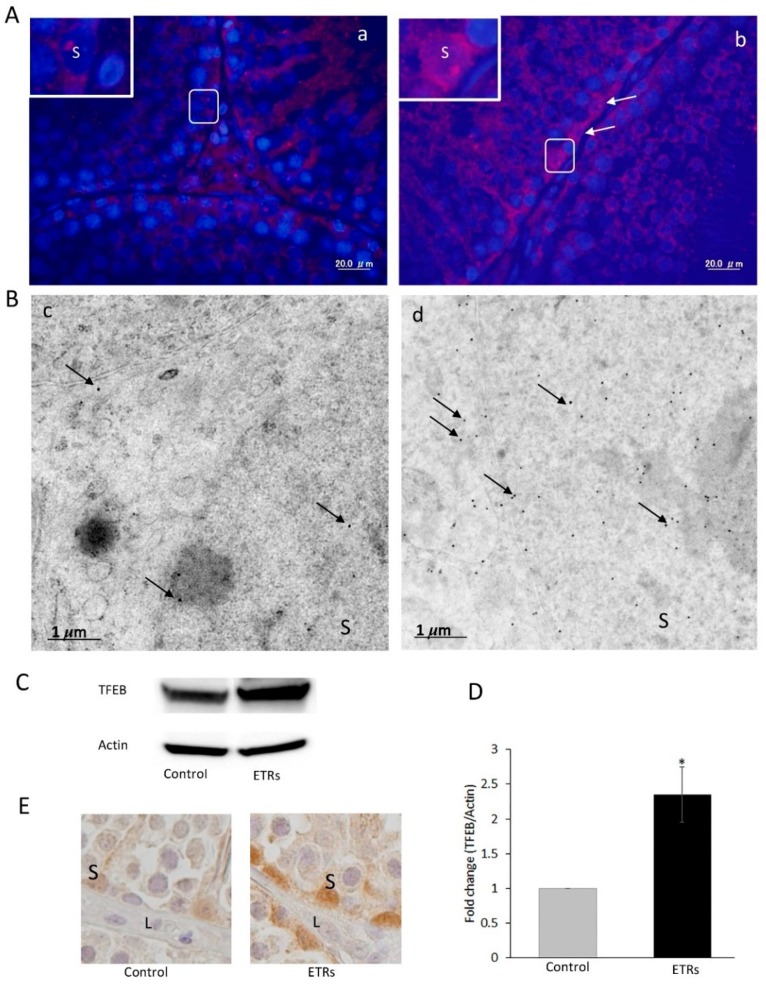
Elevated expression and nuclear translocation of TFEB in ETR SCs. (**A**) IF of TFEB expression in the control (**a**) and ETRs (**b**). The insets are higher magnifications of the framed areas. Note the overexpression of TFEB (white arrows) in SC nuclei of ETRs. (**B**) Immunogold labeling of TFEB (black arrows, 15 nm gold particles) in the control (**c**) and ETR SCs (**d**). (**C**) Western blot of TFEB in the control and ETR testes (*n* = 3). (**D**) Histogram showing a significant increase of TFEB expression in ETR testes. * *p* < 0.05 (*t*-test). (**E**) IHC showing TFEB nuclear translocation in ETR SCs (part of a seminiferous tubule), confirming the IF and IEM results (**A**,**B**). S: SC nucleus; L: Leydig cell.

**Figure 9:**
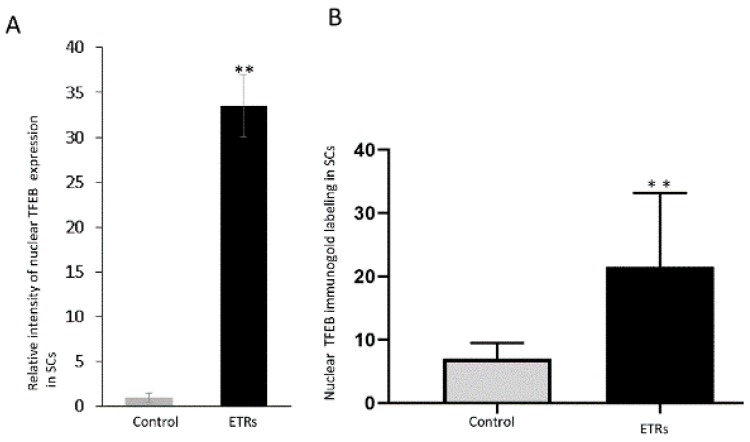
Quantitative analysis of nuclear TFEB expression and immunogold labeling in SCs. (**A**) Histogram showing significant increase of TFEB nuclear expression in ETRs SCs. (**B**) Quantification of nuclear TFEB immunogold particles in SCs. ** *p* < 0.01 (*t*-test).

**Figure 10:**
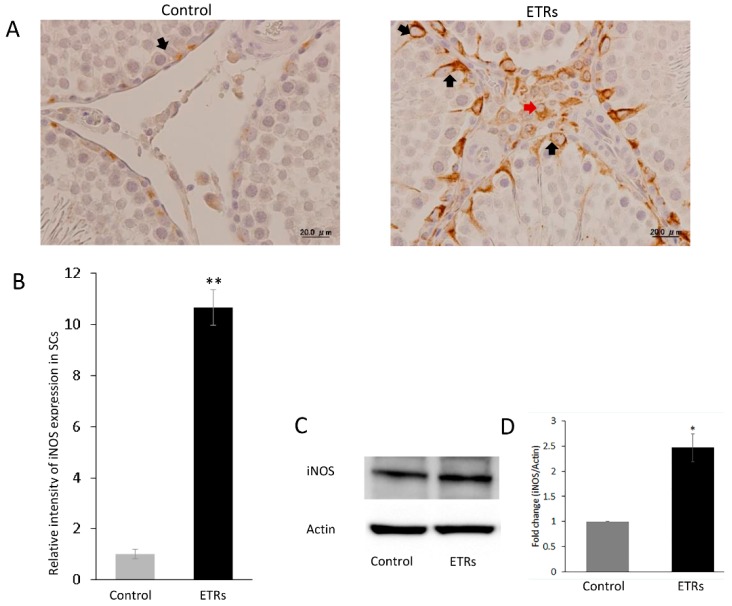
Upregulation of iNOS in ETR SCs. (**A**) IHC of iNOS in control and ETRs. Note the enhanced expression in SCs (black arrows) and interstitial cells (red arrows) of ETRs. (**B**) Quantification of iNOS expression in SCs. ** *p* < 0.01 (*t*-test). (**C**) Western blot of iNOS expression in control and ETR testes (*n* = 3). (**D**) Histogram demonstrating higher iNOS expression in ETR testes compared to the control. * *p* < 0.05 (t-test).

TFEB nuclear translocation is essential for upregulation of autophagy and mitophagy proteins upon cellular exposure to acute ethanol exposure and other producers of oxidative/nitrosative stress [16,17,18]. The authors’ investigation of this protein is shown in Figure 8. Compared to weak levels in the control group, enhanced expression and nuclear translocation of TFEB was observed in ETR SCs in IF (Figure 8A), IEM (Figure 8B), Western blot (53 kDa) (Figure 8C,D), and IHC (Figure 8E), indicating that the protein may mediate upregulated mitophagy in such cases. Quantification of nuclear TFEB expression (Figure 9A) and nuclear TFEB immunogold labeling (Figure 9B) in SCs demonstrated a significant increase in ETRs compared to control group. As nuclear translocation of this protein is related to oxidative/nitrosative stress, the expression of iNOS was investigated as shown in Figure 10. It can be seen that IHC (Figure 10A,B) and Western blot (Figure 10C,D) demonstrated the significant upregulation of iNOS (131 kDa) as observed in SCs and interstitial cells of ETR testes, compared to lower levels in the control group. This upregulation may be related to increased blood endotoxin levels and cytokines associated with ethanol toxicity [16].

## 4. Discussion

An accumulating body of data indicates that Parkin-mediated mitophagy is a prosurvival mechanism for clearance of damaged mitochondria in the liver and brain in response to various stressors such as acute ethanol exposure, subsequently preventing the release of proapoptotic proteins and the generation of toxic reactive oxygen species [4,6,12,13,14,32]. Although SCs play a central role in germ cell survival, fertility, and maintenance of testicular homeostasis [16,33,34], no studies on the involvement of the PINK1-Parkin pathway in the enhanced mitophagic response of SCs to acute ethanol intake have yet been reported. The novel findings of the current study include enhanced mitophagic response in ETR SCs, which are associated with Parkin mitochondrial translocation and colocalization with lysosome, formation of LC3-II-decorated mitophagosomes associated with reduction of mitochondrial proteins, and nuclear translocation of TFEB. These findings are based on various methods including TEM, TUNEL, IEM, IHC, IF, and Western blot. This enhanced Parkin-related mitophagy in ETR SCs seems to be a protective mechanism and may have therapeutic implications for male fertility.

The TEM findings relating to enhanced mitochondrial damage and MV formation in ETR SCs in the current study are consistent with the authors’ previous studies [6,7,8,9]. While these SCs were loaded with damaged mitochondria and MVs, they appeared normal based on TEM and TUNEL. In addition, the specific juxtanuclear accumulation of damaged mitochondria and MVs in ETR SCs indicates that the PINK1-Parkin pathway may mediate this accumulation and activate mitophagy [6,7,10,11]. Enhanced autophagic clearance of damaged mitochondria within MVs of ETR SCs in the current study is evidenced by engulfment of these mitochondria by LC3-II-decorated autophagosomal membranes [26], enhanced co-localization of LC3 with cytochrome c and p62, and reduced cytochrome c protein levels [6,10,11,23,24,31,35]. 

A major finding of the current study was Parkin overexpression and mitochondrial translocation in ETR SCs. This is supported by the authors’ previous studies on rat liver modeling of acute ethanol exposure [6,7]. However, in the current study, IEM results demonstrated the co-overexpression of PINK1 and Parkin in damaged mitochondria and mitophagosomes of ETR SCs. This overexpression may cause a collapse in the normal tubular mitochondrial network, resulting in perinuclear mitochondrial fragmentation and MV formation as previously reported [10,11]. PINK1 and Parkin mitochondrial translocation in ETR SCs in the current study may have been induced by nitrosative/oxidative stress, mt DNA damage (as evidenced by damaged cristae and matrix elements), mitochondrial depolarization, and other mechanisms [6,7,11,12,35,36]. In addition, Parkin-labeled mitochondria within mitophagosomes in ETR SCs were targeted as lysosomes for degradation based on enhanced colocalization of pan-cathepsin with Parkin and LC3-II, indicating enhanced autolysosome or mitophagolysosome formation [6,11,16,37]. In fact, autophagy is considered essential for Parkin-mediated mitochondrial clearance, as knocking out ATG5 cells resulted in failure to eliminate damaged mitochondria even with Parkin recruitment [38]. 

The current study showed that the very low Parkin-related mitophagy observed in control SCs was upregulated by ethanol in SCs, indicating that this pathway is mainly activated in response to stress signals [36]. However, it is not possible to rule out the presence of PINK1-Parkin independent mitophagic mechanisms such as mitophagy with the receptors NIX, BNIP3, and FUNDC1 and prohibitins involved in enhanced mitophagy in ETR SCs [6,7,12,13,39]. In addition, Parkin upregulation in SCs of ETRs may have other functions beyond mitophagy such as mtDNA repair, selective escape of antiapoptotic proteins from mitochondria to the endoplasmic reticulum during mitophagy, and suppression of mitochondrial spheroid formation [6,7,31].

Another important finding of the current work was the detection, using light/electronic microscopic techniques and Western blot, of the overexpression and nuclear translocation of TFEB in ETR SCs. This translocation has been reported as necessary for the upregulation of autophagy and mitophagy proteins, specifically under oxidative stress [18]. The current study results indicate that such translocation may be related to ethanol-induced oxidative/nitrosative stress as evidenced by upregulation of iNOS [16,17,18]. A recent study revealed that mitophagy-induced translocation of TFEB to the nucleus requires both PINK1 and Parkin, in contrast with starvation-induced TFEB translocation, suggesting that mitochondria and lysosomes impact each other [40].

The enhanced mitophagic response in SCs of ETRs in the current study may be anti-apoptotic mechanism [6,12,13,16,41], and may suppress the inflammatory response related to mitochondrial damage, thus maintaining testicular homeostasis [42]. In addition, it may provide lactate for germ cells via a Warburg-like effect via catabolism of damaged mitochondria [33,34,43]. Pharmacological activation of PINK1-Parkin-related mitophagy has been reported to improve mitochondrial function in animal models of Alzheimer’s disease [44,45], while overexpression of Parkin has been reported to protect retinal ganglion cells in an experimental glaucoma rat model [15] and to improve cardiac function in rats with myocardial infarction [46] via activation of mitophagy. Accordingly, Parkin-related therapy may have implications in testicular diseases and infertility associated with mitochondrial dysfunction. In addition, selective stimulation of Parkin-mediated mitophagy via the enhancement of its expression and/or mitochondrial translocation using natural or pharmaceutical products may improve mitochondrial quality as reported in relation to alcoholic liver and neurodegenerative diseases such as PD [7,45].

## 5. Conclusions

The results of this study showed morphological and molecular evidence for ethanol-induced mitophagy in SCs as represented by activation of the PINK1-Parkin pathway and nuclear translocation of TFEB, which may be mediated by oxidative stress.

## Supplementary Materials

The following are available online at https://www.mdpi.com/2073-4409/8/3/283/s1. Figure S1: Low-power micrograph showing perinuclear accumulation of damaged mitochondria (broken arrows) and mitophagic vacuoles (arrows) in SC of ETRs. S: SC nucleus; LD: lipid droplet. Figure S2: TUNEL positive germ cells (green labeling) of ETRs with non-apoptotic. Arrows mark SCs nuclei. Figure S3: Enhanced LC3 (red) and p62 (green) colocalization in SCs of ETRs. The white arrows indicated colocalization signals upon merging (yellow-orange). Figure S4: Overexpression of PINK1 in ETR SCs. Arrows indicate SCs nuclei. Figure S5: IF double labeling of LC3 (red) and Pan cathepsin (green) in ETRs. Arrows mark enhanced colocalization signals (yellow-orange) in SCs. S, SC nucleus.
