# Supplementary material for: Ethanol-Induced Mitochondrial Damage in Sertoli Cells is Associated with Parkin Overexpression and Activation of Mitophagy
Source: Cells. 2019 Mar 25;8(3):283. doi: 10.3390/cells8030283 (PMC6468925; doi:10.3390/cells8030283)
Supplement: Supplementary file 1 [file cells-08-00283-s001.pdf]

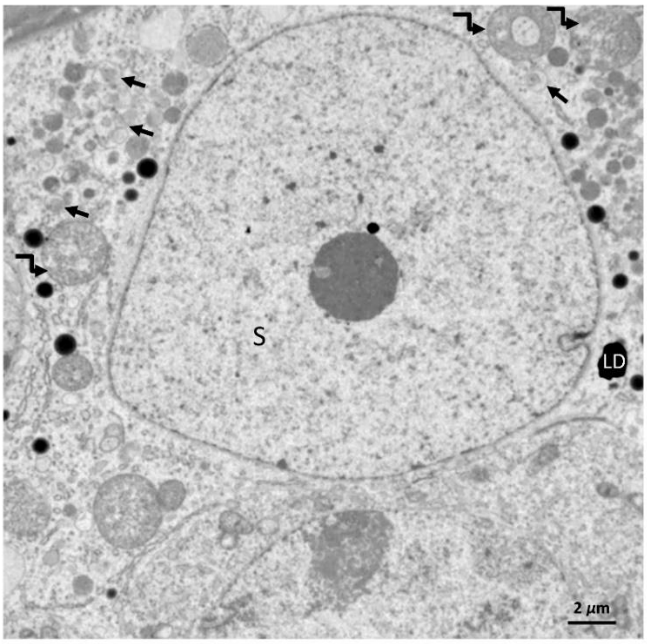

Figure S1: Low-power micrograph showing perinuclear accumulation of damaged mitochondria (broken arrows) and mitophagic vacuoles (arrows) in SC of ETRs. S: SC nucleus; LD: lipid droplet**.**

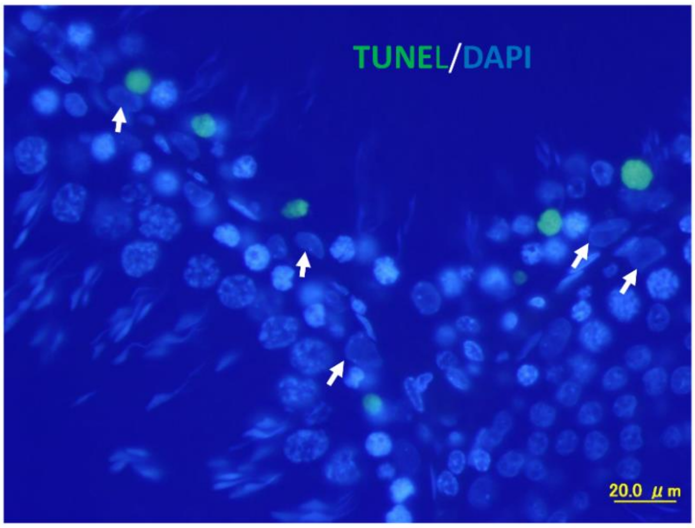

Figure S2: TUNEL positive germ cells (green labeling) of ETRs with non-apoptotic. Arrows mark SCs nuclei.

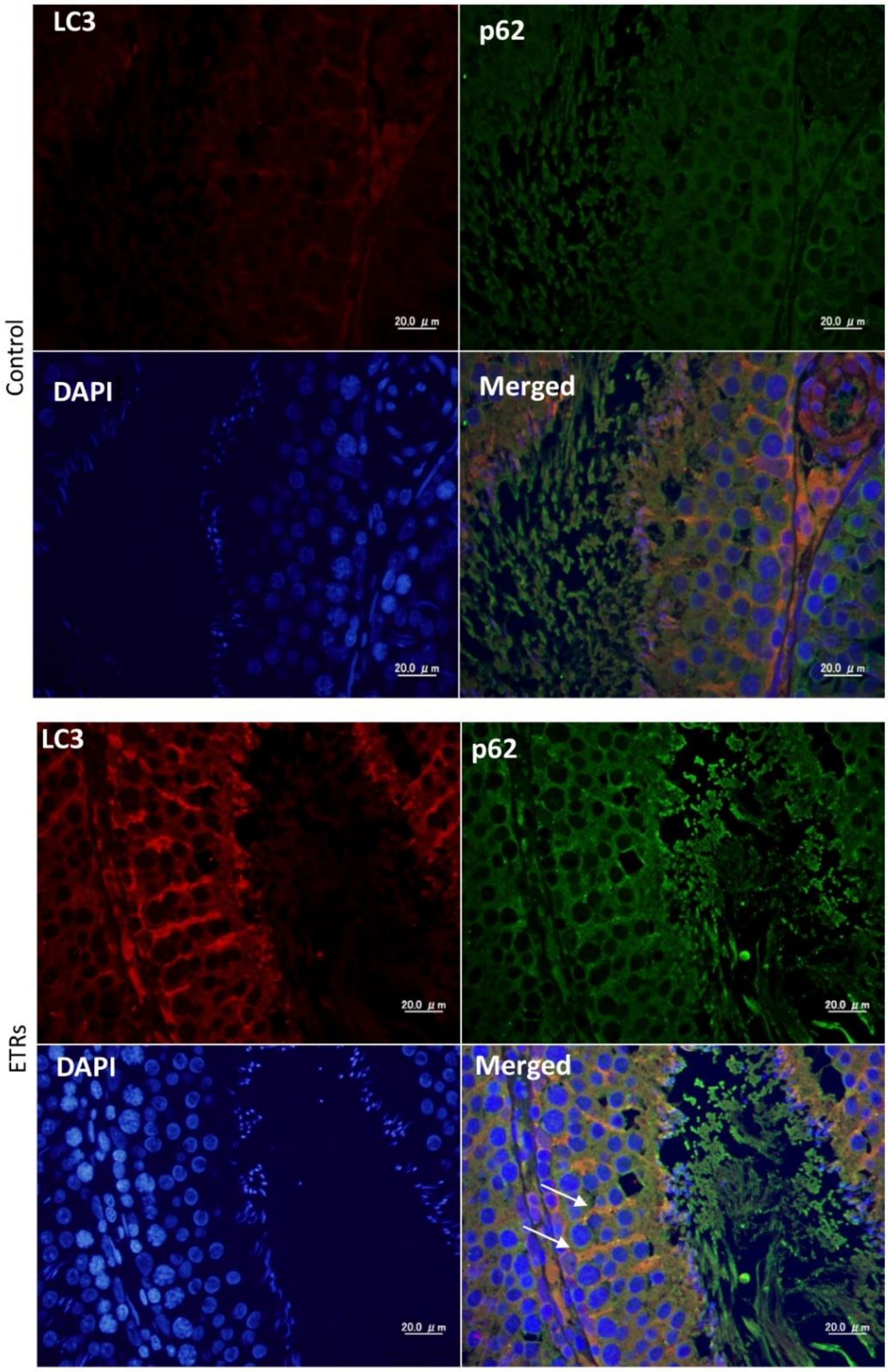

Figure S3. Enhanced LC3 (red) and p62 (green) colocalization in SCs of ETRs. The white arrows indicated colocalization signals upon merging (yellow-orange).

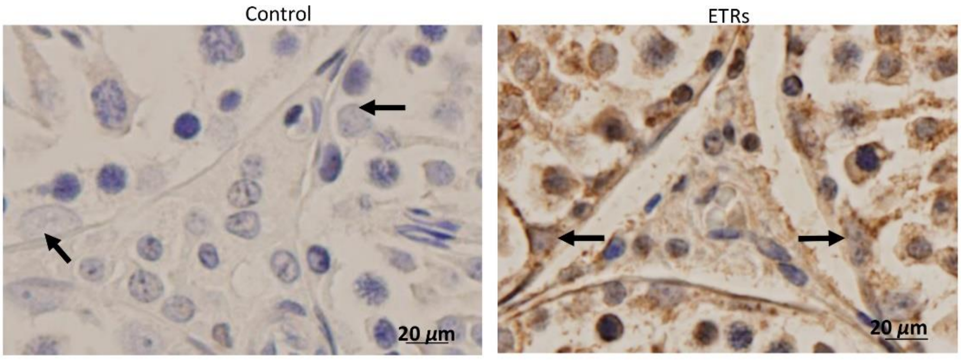

Figure S4: Overexpression of PINK1 in ETR SCs. Arrows indicate SCs nuclei.

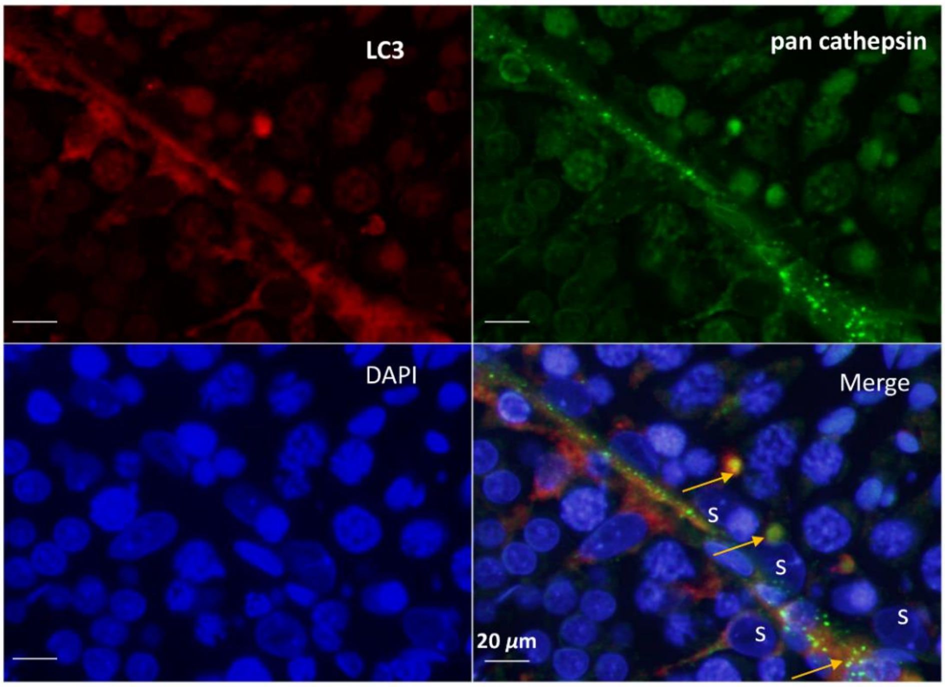

Figure S5: IF double labeling of LC3 (red) and Pan cathepsin (green) in ETRs. Arrows mark enhanced colocalization signals (yellow-orange) in SCs. S, SC nucleus.
